# The role of AKR1 family in tamoxifen resistant invasive lobular breast cancer based on data mining

**DOI:** 10.1186/s12885-021-09040-8

**Published:** 2021-12-09

**Authors:** Dong Xu, Yiqi Zhang, Feng Jin

**Affiliations:** Department of Breast Surgery, The First Affiliated Hospital of China Medical University, 155N Nanjing Street, Heping, Shenyang, 110001 Liaoning China

**Keywords:** Invasive lobular breast cancer, Tamoxifen, Aldo-keto reductase family 1, Data mining, Differentially expressed genes

## Abstract

**Background:**

Tamoxifen (TAM) resistance to invasive lobular cell carcinoma is a challenge for breast cancer treatment. This study explored the role of Aldo-keto reductase family 1 (AKR1) family in tamoxifen-resistant aggressive lobular breast cancer based on data mining.

**Methods:**

TAM-resistant invasive lobular breast cancer gene chip was downloaded from the Gene Expression Omnibus (GEO) database (accession-numbered as GSE96670). The online analytical tool GEO2R was used to screen for differentially expressed genes in TAM-resistant invasive lobular breast cancer cells and TAM-sensitive counterparts. A protein-protein interaction (PPI) networks were constructed using the STRING online platform and the Cytoscape software. GeneMANIA and GSCALite online tools were used to reveal the potential role of these hub genes in breast cancer progression and TAM resistance development. And the used the GSE67916 microarray data set to verify the differentially expression of these hub genes in breast cancer. The protein expression levels of *AKR1C1*, *AKR1C2* and *AKR1C3* in TAM-sensitive and resistant breast cancer cells were compared. The TAM sensitivity of breast cancer cells with or without *AKR1C1*, *AKR1C2* or *AKR1C3* gene manipulation was evaluated by cell viability assay.

**Results:**

A total of 184 differentially expressed genes were screened. Compared with TAM sensitive breast cancer cells, 162 were up-regulated and 22 were down-regulated. The study identified several hub genes in the PPI network that may be involved in the development of TAM resistance of breast cancer, including signal transducer and activator of transcription 1 (*STAT1*), estrogen receptor alpha (*ESR1*), fibronectin1 (*FN1*), cytochrome P4501B1 (*CYP1B1*), *AKR1C1*, *AKR1C2*, *AKR1C3* and uridine diphosphate glucuronosyltransferase (*UGT*) 1A family genes (*UGT1A1*, *UGT1A*3, *UGT1A4*, *UGT1A6*, *UGT1A7*, *UGT1A8*, *UGT1A9*, *UGT1A10*). Compared with TAM-sensitive counterparts, the expression levels of *AKR1C1*, *AKR1C2*, and *AKR1C3* were up-regulated in TAM-resistant breast cancer cells.

**Conclusions:**

Overexpression of each of these three genes significantly increased the resistance of breast cancer cells to TAM treatment, while their knockdown showed opposite effects, indicating that they are potential therapeutic target for the treatment of TAM-resistant breast cancer.

**Supplementary Information:**

The online version contains supplementary material available at 10.1186/s12885-021-09040-8.

## Background

The incidence of breast cancer is increasing gradually recently, which seriously threatens the life and health of women [1]. Invasive lobular breast cancer is one of the common pathological types of breast cancer, which is second only to invasive ductal cancer, with strong metastasis and invasion, and it has a high mortality and recurrence rate [2]. The occurrence and development of breast cancer is influenced by many factors, but the mechanism is not clear at present [3, 4]. Tamoxifen (TAM) is a classic drug for endocrine therapy of breast cancer, especially for estrogen receptor (ER) positive patients with better effect and longer duration, which can significantly reduce the recurrence rate and mortality of tumor [5]. However, data shows that 40% of breast cancer patients develop TAM resistance during initial treatment, and 25% of patients receive effective treatment at the initial stage, and it is easy to develop drug resistance after a period of time [6]. And the resistance of breast cancer to TAM limits its clinical therapeutic effect [7, 8]. TAM resistance is more complicated. It is currently believed to be related to inactivation of tumor suppressor genes, activation of oncogenes, and abnormal expression of ER, but the specific mechanism is still elusive [9].

Bioinformatics is widely used in the medical field. For example, Bioinformatics software can be used for key gene screening, experimental design, disease diagnosis and proteomics research. Recently, genomic DNA copy number arrays, messenger RNA arrays, exon sequencing, DNA methylation, microRNA sequencing, and protein arrays are used to clarify the subtype and molecular mechanism of breast cancer. Datasets are deposited in public databases, such as the cancer genome atlas (TCGA), and these data approve the heterogeneity of clinical behavior [10]. Besides, these public datasets provide the possibility to investigate the molecular mechanism from different perspectives. Thus, an in-depth understanding of the molecular pattern of breast cancer can help formulate new strategies for the treatment of cancer [11]. In this research, we identified genes differentially expressed genes in TAM-resistant invasive lobular breast cancer cells and TAM-sensitive counterpart, and used STRING online tool to reconstruct the protein interaction relationship network among these genes, from which we located some genes at the network nodes. Then we used GeneMANIA and GSCALite databases to analyze the signal pathways and tumor resistance that these node genes may be involved in, and compared the expression differences of these genes between TAM-resistant and TAM-sensitive breast cancer cells in the GSE67916 microarray dataset. We found that compared with TAM-sensitive breast cancer cells, the expression levels of *AKR1C1*, *AKR1C2* and *AKR1C3* genes were significantly increased in TAM-resistant breast cancer cells. The function of *AKR1C1*, *AKR1C2* and *AKR1C3* genes in TAM-resistant breast cancer cells was revealed by bioinformatics analysis and further confirmed by biological experiments.

## Materials and methods

### Screening of differentially expressed genes

The microarray data included 12 samples, such as TAM-sensitive invasive lobular breast cancer cell line (SUM44), TAM-resistant invasive lobular breast cancer cell line (LCCTam), TAM-treated invasive lobular breast cancer cell line (SUM44-4HT) for 24 h and TAM-deficient cell line treated for 14 d, 3 repetition was performed for each sample. In addition, download the microarray data GSE67916 as the subsequent differential expression gene verification chip, and the platform used was GPL570 [HG-U133_Plus_2] Affymetrix Human Genome U133 Plus 2.0 Array. It included 18 samples, the first 10 samples were TAM-resistant cell lines (TamR), and the last 8 were TAM-sensitive cell lines (MCF7/S0.5). GEO2R (https://www.ncbi.nlm.nih.Gov/geo/geo2r/) is an online analysis tool of Gene expression omnibus (GEO), which can analyze the gene differential expression of some GEO samples. According to GEO2R setting conditions: adj. P.Val < 0.05, and log2 (FC) absolute value > 1. We first used the GEO2R to compare SUM44 and LCCTam, and screen out the possible differentially expressed genes for drug resistance. Then compared the invasive lobular breast cancer cell line (SUM44-4HT) and SUM44 treated with TAM for 24 h, and screened out possible differentially expressed genes that were involved in early TAM resistance. The volcano plot of differentially expressed genes was made by Chris Lou’s online website (http://www.chrislifescience.club:3838/R/AnnoE2/). The function of Venn diagram in the local tool funrich software was used to get the intersection of these differentially expressed genes. The heat map of the differentially expressed genes after intersection was made by TBtools.

### Reconstruction of protein-protein interaction network and identification of hub genes

String online platform was used to obtain protein-protein interactions corresponding to the differentially expressed genes determined using the default settings. STRING (https://string-db.org/) is to collect, grade and integrate all publicly available Protein-protein interactions (PPI) data, and supplement these data by calculating and predicting potential functions [12]. The text-mining channel, STRING performs statistical co-citation analysis across on a large number of scientific texts [13]. The protein-protein interaction network was re-constructed by Cytoscape software (Ver 3.6.0), and the hub genes with a degree value > 17 were selected by MCODE plugin for the following analysis. The Cytoscape software is designed to analyze and visualize very large networks, and provides greater flexibility in importing additional data and visualizing these data to the network [14]. The PPI network complex of these DEGs was constructed by Cytoscape software, the molecular complex detection (MCODE) plug-in and the online database STRING [15]. In this study, STRING online analysis tool was used to obtain the information about the interaction between different genes, and PPI file was imported into the Cytoscape software (version 3.6.0) to draw the PPI network diagram. Then the key nodes of PPI network were obtained.

### Functional analysis and drug sensitivity analysis of hub gene

Genemania (http://www.genemania.org) is a flexible and user-friendly online software, which can be used to build PPI network, generate hub gene function analysis list and sort [16]. The website has a variety of bioinformatics methods such as physical interaction, gene co expression, gene co location, gene enrichment analysis and website prediction [17]. Gene Set Cancer Analysis (GSCALite, http://bioinfo.life.hust.edu.cn/web/gscalite/) is an online genomic cancer analysis platform, which integrates cancer genome data from TCGA 33 cancer types, drug response data from GDSC and CTRP, as well as normal tissue data from GTEx, and perform gene set analysis in a unified data analysis process [6]. GSCALite to analyze a set of genes in cancers with the following functional modules. (i) Differential expression and the survival analysis between tumor and normal, (ii) Genomic variations and their survival analysis, (iii) Cancer pathway activity related to gene expression, (iv) Gene miRNA regulatory network, (v) Gene drug sensitivity, (vi) Normal tissue expression and gene eQTL. In this study, GeneMANIA and GSCALite were used to analyze the relationship between hub gene and drug resistance mechanism and drug sensitivity.

### Cell culture

TAM-sensitive cell line and TMA-resistant breast cancer cells are gifts from Dr. Clarke in Georgetown University Medical Center. 10% fetal bovine serum (FBS) purchased from Sigma-Aldrich. Cells were treated by DMEM/F12 medium containing 10% FBS. Overexpressing empty vectors (pcDNA-NC), overexpressing AKR1C1 vectors (pcDNA-AKR1C1), overexpressing AKR1C2 vectors (pcDNA-AKR1C2), overexpressing AKR1C3 vectors (pcDNA-AKR1C3), RNA interferes with empty plasmids (pSUPER-si-NC), RNA interferes with AKR1C1 plasmids (pSUPER-si-AKR1C1), RNA interferes with AKR1C2 plasmids (pSUPER-si-AKR1C2), RNA interferes with AKR1C3 plasmids (pSUPER-si-AKR1C3) were constructed by Shanghai Sangon Biological Co. LTD. The naive cell line (TAM-sensitive) transfected with pcDNA-NC, pcDNA-AKR1C1, pcDNA-AKR1C2 or pcDNA-AKR1C3 and the TAM-resistant cell line transfected with pSUPER-si-NC, pSUPER-si-AKR1C1, pSUPER-si-AKR1C2 or pSUPER-si-AKR1C3.

### Western blot

After 24 h of transfection, the cells of each group were collected. Cells were lysed with RIPA buffer containing protease imhibitory cocktail. Protein concentration was measured by the protein test kit, 40 μg protein samples were separated on SDS-PAGE. Then, proteins were transferred to activated with PVDF membranes using wet tank blotting. After blocking with 5% defatted milk in TBST for 2 h at room temperature, primary antibody incubations were carried out overnight at 4 °C. The secondary antibodies (1:2000) were directed against the host of primery antibodies and incubated for 2 h at room temperature. The GAPDH was the internal reference protein. The primary antibodies used for the analysis were anti-AKR1C1 (1:1000), anti-AKR1C2 (1:1000), anti-AKR1C3 (1:1000), and anti-GAPDH (1:5000) antibodies. Goat anti-mouse/rabbit double antibodies were used as the secondary antibodies. The enhanced chemiluminescence (ECL) method was used for coloration and radiography. The ImageJ software was used to observe and analyze.

### Cell proliferation test

The cells of each group were collected after 24 h of transfection. A cell counting kit-8 (CCK-8) was used to measure cell viability. Cell susppensions (1 × 10^4^ cells/ml) were seeded in 96-well plants with 100 μL DMEM/F12. After adherence, the cells were treateed with different doses of TAM, and then incubated at 37 °C for additional 48 h. After the cells were inoculated with 10% CCK-8 solution for 2 h. Then the absorbance at 450 nm was measured using a microplate reader. TMA’s half maximal inhibitory concentration (EC50) was calculated based on dose-response curve.

### Statistical analysis

Continuous data was expressed as mean ± standard deviation($\overline{x}\pm s$) and analyzed by SPSS 22.0 software. Single-factor analysis of variance was used for the comparison between groups, and *SNK-q* test was used for further pair-wise comparison. When *P* < 0.05, the difference was statistically significant.

## Results

### Screening of differentially expressed genes

**Fig. 1:**
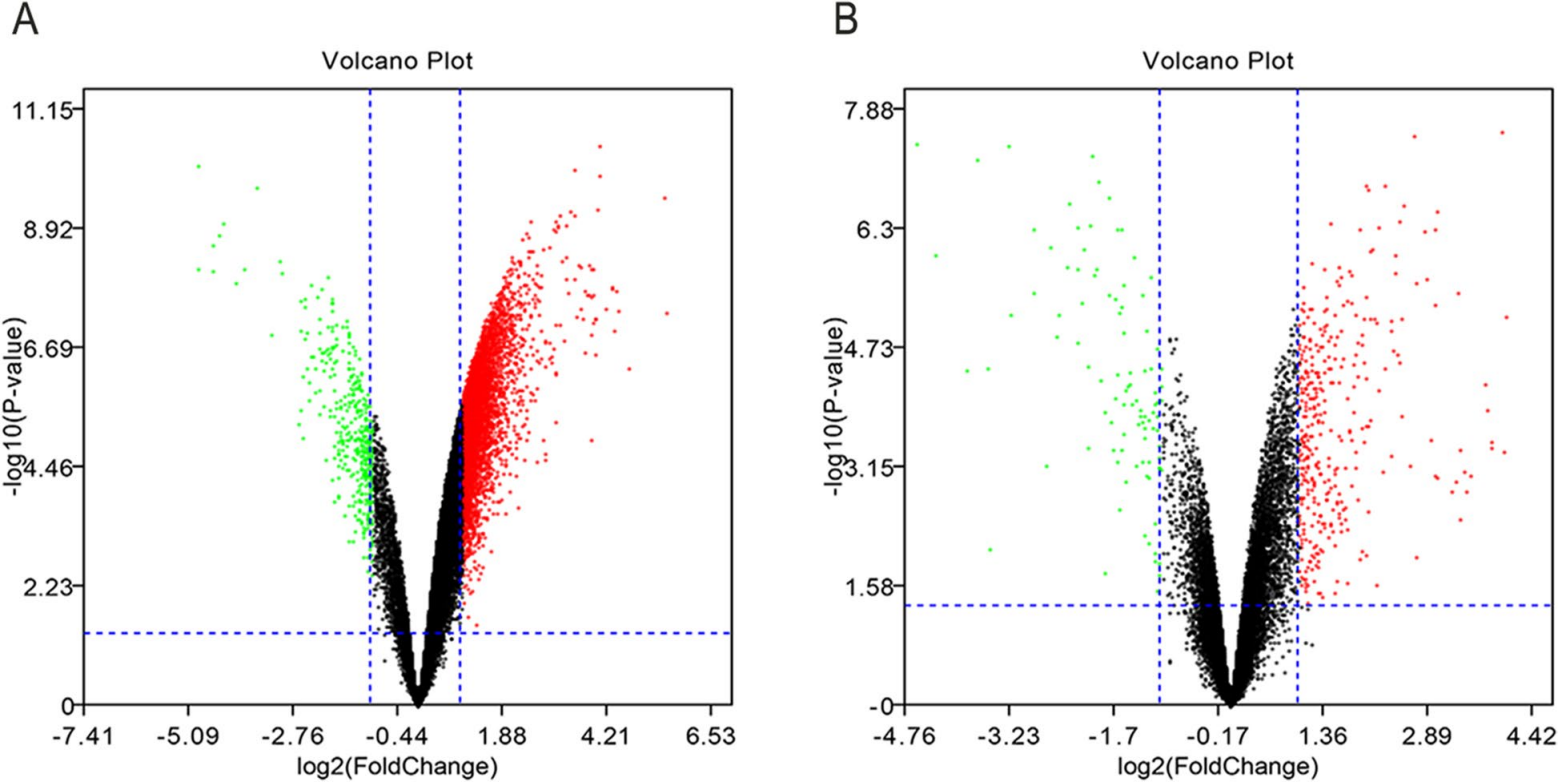
Volcano plot demonstrating the differentially expressed genes between TAM-resistance and sensitive breast cancer cells. **a** Comparison between LCCTam group and SUM44 group. **b** Comparison between SUM44-4HT group and SUM44 group. SUM44, TAM sensitive invasive lobular breast cancer cell line; LCCTam, TAM resistant invasive lobular breast cancer cell line; SUM44-4HT, TAM 24 h sensitive invasive lobular breast cancer cell line (supplement 1)

A total of 4066 significantly differentially expressed genes were screened out by comparison between LCCTam group and SUM44 group, 3719 of which were up-regulated and 347 of which were down-regulated (Fig. 1a). A total of 361 differentially expressed genes were screened out by comparison between SUM44-4HT group and SUM44 group, 283 of which were up-regulated and 78 of which were down-regulated (Fig. 1b).

### Analysis of differential expression genes of TAM resistance in early stage of invasive lobular breast cancer by Venn diagram

**Fig. 2:**
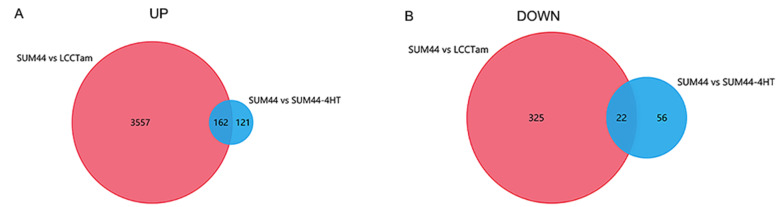
Analysis of the differential expression genes of TAM resistance in early stage of invasive lobular breast cancer by Venn diagram. **a** Up-regulated gene; **b** Down-regulated gene. *P* < 0.05, [FC] > 1 was considered as the cutoff value. SUM44, TAM sensitive invasive lobular breast cancer cell line; LCCTam, TAM resistant invasive lobular breast cancer cell line; SUM44-4HT, TAM 24 h sensitive invasive lobular breast cancer cell line

**Fig. 3:**
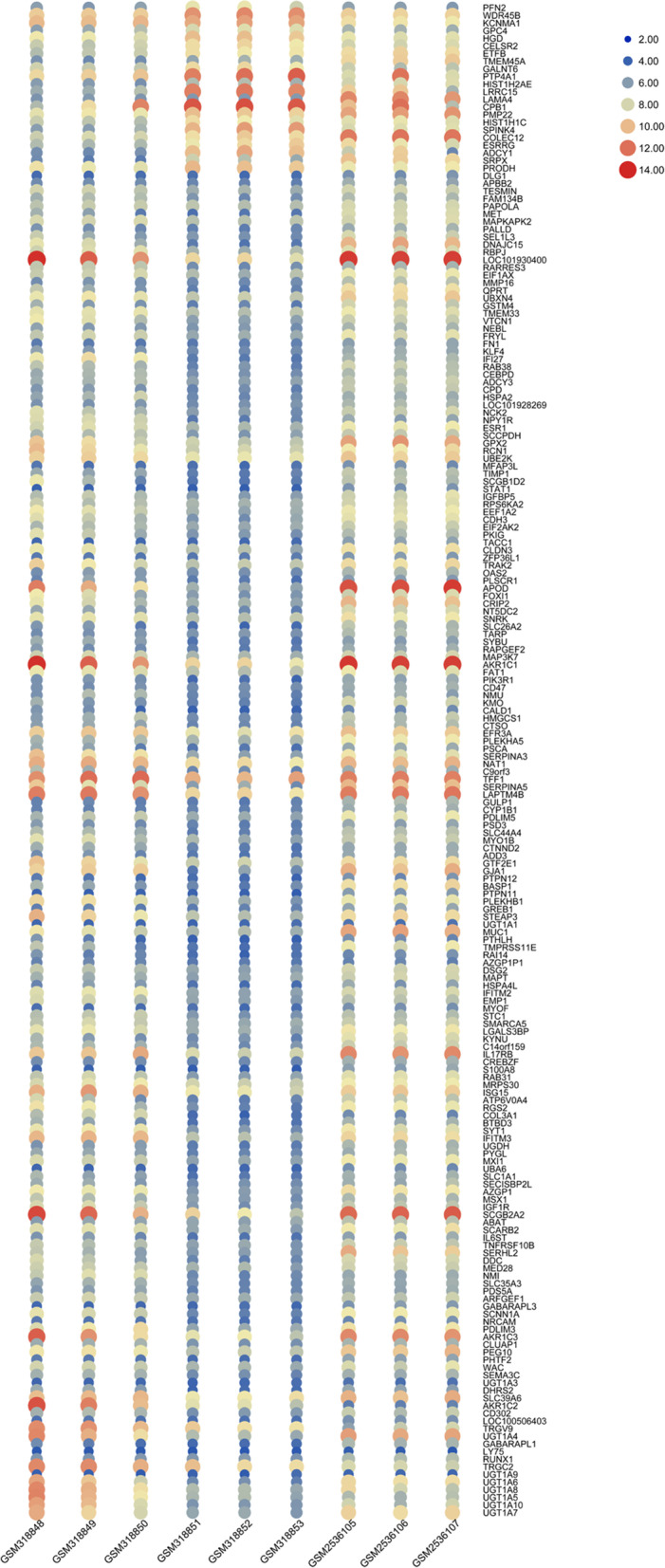
Heat map of the differentially expressed genes. Each column represented a sample (GSM318848, GSM318849 and GSM318850 belongs to SUM44; GSM318851, GSM318852 and GSM318853 belongs to LCCTam; GSM2536105, GSM2536106 and GSM2536107 belongs to SUM44-4HT), each row represented a gene, and from blue to red represented the change of genes from down regulation to up regulation

Venn diagram analysis showed that 184 significantly different expression genes were intersected in the two groups, including 162 up-regulated genes (Fig. 2a) and 22 down-regulated genes (Fig. 2b). The expression of different expression genes was shown in Fig. 3.

### Construction of PPI network and analysis of hub gene

**Fig. 4:**
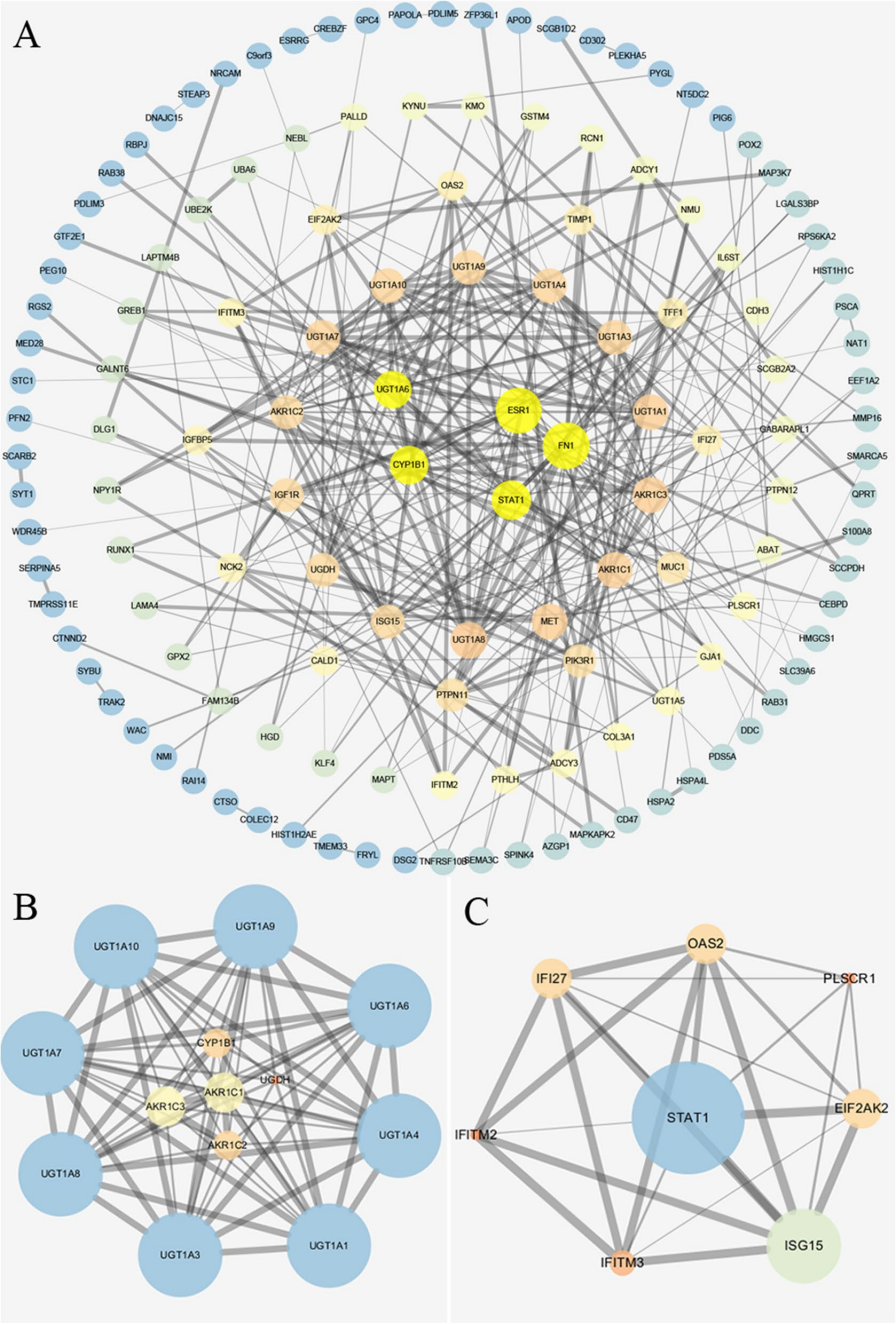
PPI network and subnetwork of the differentially expressed genes. **a** PPI network diagram of the differentially expressed genes, each ring from outside to inside represented different degrees, 1-5, 6-10, 11-15, 16-20, 21-30 in turn, gray horizontal lines represented the correlation between the genes of each node, the more lines, the closer the relationship; **b**, **c** The module 1 (score = 12.167, nodes = 13) and module 2 (score = 7.143, nodes = 8) calculated through the MCODE plug-in; the larger the degree, the larger the circular area

Using STRING on-line data analysis tool to import the selected differential expression gene into the protein interaction map, and then import it into Cytoscape for analysis and calculation. A ring graph was made according to the node degree, when the node degree value was more than 15, signal transducer and activator of transcription 1 (*STAT1*), uridine diphosphate glucuronosyltransferase (*UGT1A6*), estrogen receptor alpha (*ESR1*), fibronectin1 (*FN1*) and cytochrome P4501B1 (*CYP1B1*) were the key nodes in the PPI module (Fig. 4a). Furthermore, two subnets were obtained by MCODE plug-in. According to the degree layout sequence, the hub genes *STAT1*, *ESR1*, *FN1*, *CYP1B1*, *AKR1C1*, *AKR1C2*, *AKR1C3* and UGT1A family genes (*UGT1A1*, *UGT1A3*, *UGT1A4*, *UGT1A6*, *UGT1A7*, *UGT1A8*, *UGT1A9*, *UGT1A10*) were screened out (Fig. 4b, c).

### Functional analysis of hub gene

**Fig. 5:**
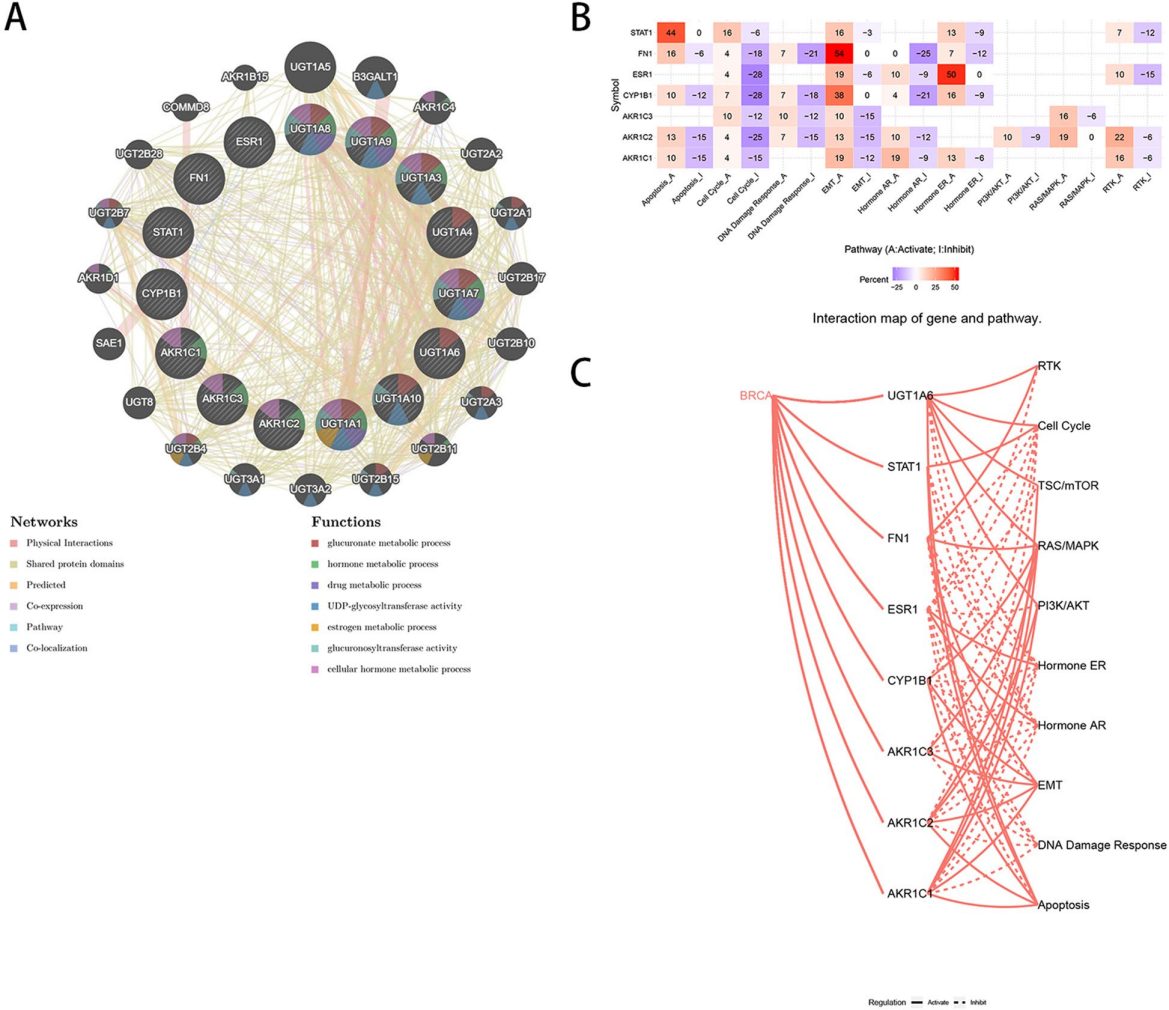
PPI network and function analysis of hub gene. **a** GeneMANIA software constructs PPI network map of hub gene, and different colors represent different pathways involved; **b**, **c** GSCALite software analyzed the pathway map related to the mechanism of hub gene resistance, and the larger the number in **b** diagram represented the stronger the correlation, and the sign represented inhibition and promotion respectively

In order to further analyze the function of the selected hub gene in TAM resistance, we imported the hub gene into the GeneMANIA database. The analysis results showed that *UGT1A1*, *UGT1A3*, *UGT1A7*, *UGT1A8*, *UGT1A9*, *AKR1C1*, *AKR1C2*, *AKR1C3* and other genes were related to glucuronic acid metabolism process, drug metabolism process, UDP-glycosyltransferase activity, hormone metabolism and cell hormone metabolism function (Fig. 5a). GSCALite software was used to annotate gene model pathways. GSCALite database only showed that *STAT1*, *FN1*, *ESR1*, *CYP1B1*, *AKR1C1*, *AKR1C2*, *AKR1C3* and other genes were involved in the pathways and functions, mainly involving PI3K/AKT, RAS/MAPK, RTK and other pathways, as well as the processes of apoptosis, cell cycle inhibition, epithelial mesenchymal transition (EMT), estrogen receptor (ER), androgen receptor (AR) activation (Fig. 5b, c). The two databases indicated that hub gene was involved in the metabolism of estrogen and androgen, especially *AKR1C1*, *AKR1C2*, *AKR1C3*, *ESR1* and so on. It was speculated that *AKR1* family gene may affect Tam resistance by participating in estrogen and androgen metabolism.

### Resistance analysis of hub gene

**Fig. 6:**
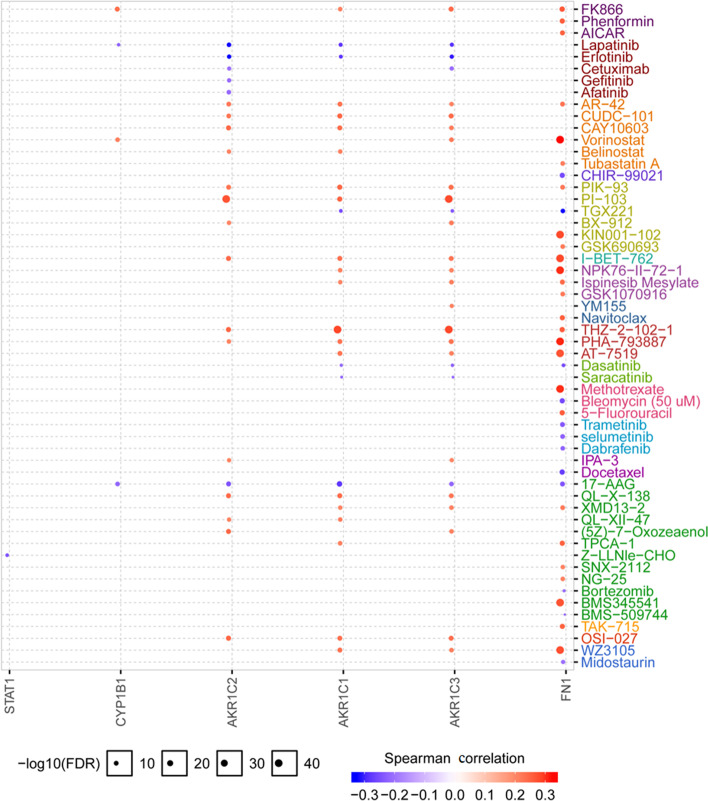
Correlation analysis of hub gene and drug sensitivity (GSCALite). Red indicated positive correlation and blue indicated negative correlation, the darker the color and the stronger the correlation, the ordinate of the circle was the corrected *P* value, the larger the circle, the greater the value, the more significant the difference

In order to verify the correlation between hub gene and TAM resistance in breast cancer, this study imported hub gene into GSCALite database for analysis, the results showed that *AKR1C1*, *AKR1C2*, *AKR1C3* and *FN1* genes were related to tyrosine kinase inhibitors, EGFR inhibitors, MEK inhibitors, proteasome inhibitors, folic acids resistant, pyrimidines resistant and other drug resistance (Fig. 6), suggesting *AKR1C1*, *AKR1C2*, *AKR1C3*, *FN1* and other genes have multidrug resistance, but the database does not include the situation of TAM resistance. According to previous experience [18], multidrug resistance genes often participate in the drug resistance process through a variety of ways. Compare with other hub genes screened, *AKR1C1*, *AKR1C2*, *AKR1C3*, *FN1* are more likely to participate in the process of TAM resistance.

### Hub gene verification

**Fig. 7:**
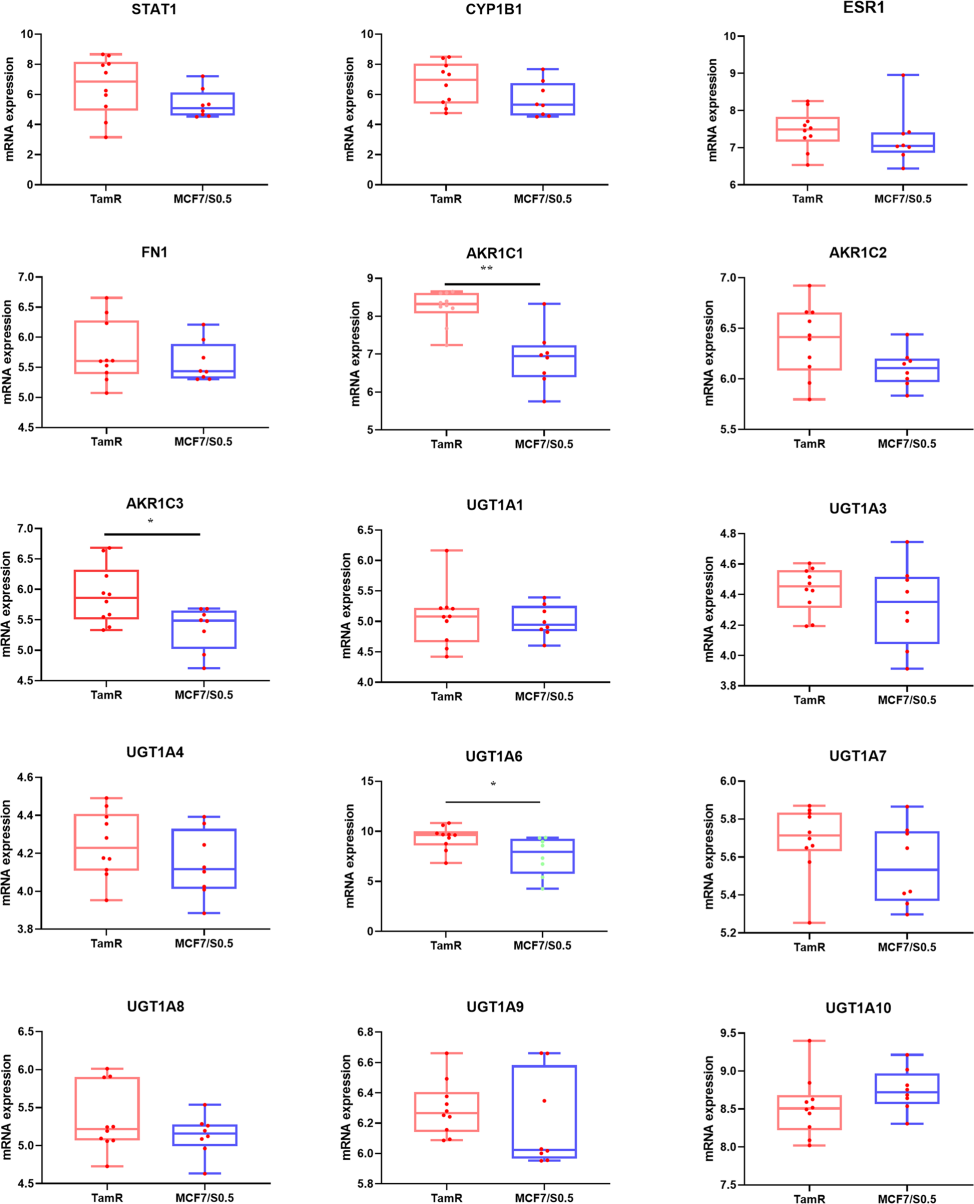
Validation of the expression differences of each gene between TAM-sensitive and resistant breast cancer cells of hub gene in microarray data GSE67916. Box line and scatter diagram were used to represent the expression of hub gene in TamR and MCF7/S0.5 groups

In order to further verify the expression difference of hub gene in TAM resistance, we download microarray data GSE67916 to verify the genes of *STAT1*, *ESR1*, *FN1*, *CYP1B1*, *AKR1C1*, *AKR1C2*, *AKR1C3* and *UGT1A* family. The data set was divided into TAM resistance group (TamR) and TAM sensitive group (MCF7/S0.5). The expression of *AKR1C1*, *AKR1C3* and *UGT1A6* in TamR group was significantly higher than that in MCF7/S0.5 group (*P* < 0.05) (Fig. 7), while there was no significant difference in the expression of other genes between the two groups. It was worth noting that the *UGT1A6* is highly expressed in the TamR group, indicating that the UDP-glucuronyl transferase encoded by UGT1A6 also played an important role in TAM-resistance invasive lobular breast cancer cells.

### Knockdown or overexpression of *AKR1C1*, *AKR1C2* or *AKR1C3* significantly affects the sensitivity of breast cancer cells to tamoxifen

**Fig. 8:**
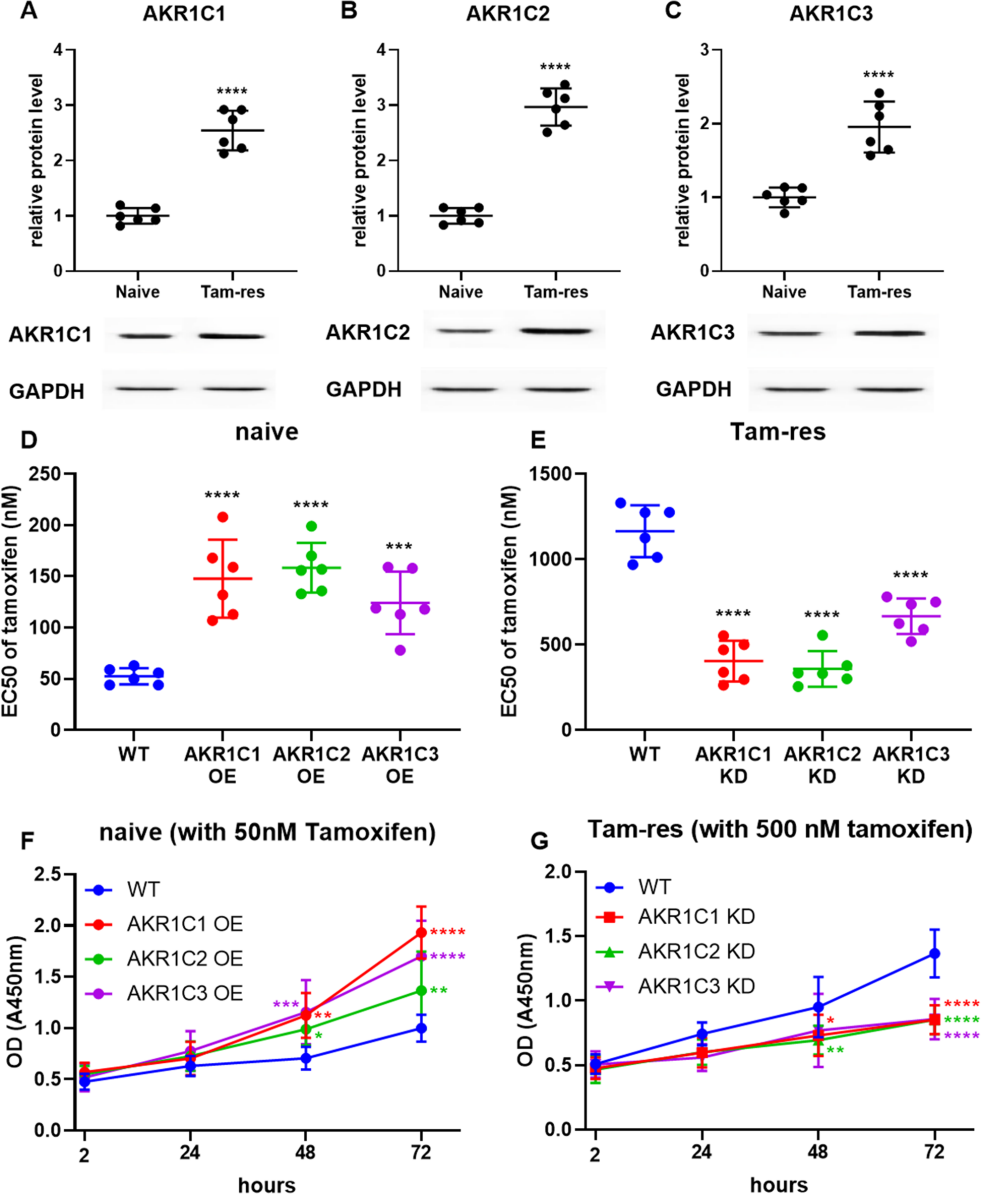
Knockdown or overexpression of AKR1C1, AKR1C2 and AKR1C3 significantly affected the sensitivity of breast cancer cells to TAM. **a**, **b**, **c** The protein levels of the three genes in the established TAM-resistant strains were significantly increased (supplement 2). **d**, **e** After overexpression or knockdown of AKR1C1, AKR1C2 or AKR1C3, significantly affected the TAM EC50 value of TAM-sensitive (naive) or resistant (Tam-res) breast cancer cells. **f**, **g** After overexpression or knockdown of AKR1C1, AKR1C2 or AKR1C3, significantly affected the cell proliferation of TAM-sensitive (naive) or resistant (Tam-res) breast cancer cells. *P < 0.05; **P < 0.01; ***P < 0.001; ****P < 0.0001

In order to analyze the *AKR1C1*, *AKR1C2* or *AKR1C3* protein expression levels in different cell lines, the western blot was used for analysis. The results showed the *AKR1C1*, *AKR1C2* and *AKR1C3* were highly expressed in TAM-resistant breast cancer cells (Fig. 8a, b, c). In addition, the CCK-8 assay results showed that in TAM-sensitive invasive lobular breast cancer cells, the overexpression of *AKR1C1*, *AKR1C2* and *AKR1C3* could significantly increase the cell TAM EC50 value (Fig. 8d), and in TAM-resistant invasive lobular breast cancer cells, knockdown of *AKR1C1*, *AKR1C2* and *AKR1C3* could significantly reduce the cell TAM EC50 value (Fig. 8e). The proliferation activity of TAM-sensitive invasive lobular breast cancer cells after overexpressing *AKR1C1*, *AKR1C2* and *AKR1C3* treated with 50 nM TMA was significantly higher than that of wild type (Fig. 8f); 500 nM TAM was used to treat TAM-resistant invasive lobular breast cancer cells, knocking out *AKR1C1*, *AKR1C2* and *AKR1C3*, its proliferation activity was significantly lower than that of wild type (Fig. 8g).

## Discussion

In this study, bioinformatics tools were used to screen the significantly differentially expressed genes in the database of different cell lines. They are TAM sensitive/resistant invasive lobular breast cancer cell lines and TAM sensitive/resistant invasive lobular breast cancer cell lines after 24 h treatment. The results showed that the former screened 4066 differentially expressed genes, of which 3719 were up-regulated and 347 down-regulated; the latter screened 361 differentially expressed genes, of which 283 were up-regulated and 78 down-regulated. In addition, Venn diagram analysis showed that 184 significantly different expression genes were intersected in the two groups,of which 162 were up-regulated and 22 were down-regulated.

Previous studies showed that the inhibition of Wnt signaling pathway was related to TAM resistance in breast cancer. Zheng et al. [19] found that vitamin D increased the sensitivity of breast cancer cells to TAM by inhibiting Wnt/β-catenin signaling pathway. Glucuronosyltransferase is a membrane linked enzyme, which plays an important role in the metabolism of exogenous substances. Hammad et al. [20] discovered that TAM induced gene knockout can increase the toxicity of mouse liver and reduce the activity of glucuronosyltransferase. The above signaling pathway and biological functions were related to the resistance of TAM. Ahn et al. [21] showed that TAM could significantly inhibit the β cell proliferation of C57BL6 gene mice, and its mechanism might be related to the inhibition of cyclin D1 and D2 RNA. Zhou et al. [22] found that Osthol could reduce the oxidative damage induced by TAM and alleviate the hepatotoxicity by increasing the levels of cAMP and cGMP in the liver. These studies had confirmed that insulin secretion and cGMP-PKG pathway were involved in the pharmacological process of TAM, which might be related to the drug resistance of TAM. Through PPI network construction and analysis, the hub genes were *STAT1*, *ESR1*, *FN1*, *CYP1B1*, *AKR1* family (*AKR1C1*, *AKR1C2*, *AKR1C3*) and *UGT1A* family genes (*UGT1A1*, *UGT1A3*, *UGT1A4*, *UGT1A6*, *UGT1A7*, *UGT1A8*, *UGT1A9*, *UGT1A10*). It has been reported that the abnormal expression of *STAT1* [23–25], *ESR1* [26, 27], *CYP1B1* [28, 29], *FN1* [30] and *UGT1A* family genes [31, 32] is closely related to breast cancer and TAM multidrug resistance, while the relationship between *AKR1* family and breast cancer and TAM resistance has not been studied. The results showed that *UGT1A1*, *UGT1A3*, *UGT1A7*, *UGT1A8*, *UGT1A9*, *AKR1C1*, *AKR1C*2, *AKR1C3* and other genes were related to glucuronic acid metabolism, drug metabolism, UDP-glycosyltransferase activity, hormone metabolism and cell hormone metabolism.

GSCALite database only showed the pathways and functions of *STAT1*, *FN1*, *ESR1*, *CYP1B1*, *AKR1C1*, *AKR1C*2, *AKR1C3* and other genes, mainly involving PI3K/AKT, RAS/MAPK, RTK and other pathways, also apoptosis, cell cycle inhibition, EMT, ER, AR activation and other processes. Both databases showed that hub genes were involved in the metabolism of estrogen and androgen, especially *AKR1C1*, *AKR1C2*, *AKR1C3*, *ESR1* and other genes. TAM has significant benefits in ERα positive breast cancer patients. The inhibition of ER expression or the loss of ER activity is related to TAM resistance, it is may be related to the mechanism by which ER mutations cause changes in ligand transcription levels to regulate breast cancer cell proliferation and induce TAM resistance [33, 34]. However, the therapeutic effect of AR/ER ratio on breast cancer has not been fully determined. It has been reported that compared with Ki67 and PgR, AR expression level has no effect on the treatment of advanced breast cancer patients with estrogen [35]. AKR1 is a 3-ketosterol reductase, which is closely related to steroid metabolism. AKR1 can reduce the level of dihydrotestosterone and prevent AR activation. AKR1C family includes *AKR1C1*, *AKR1C2*, *AKR1C3* and *AKR1C4*. Studies have shown that compared with matched benign tissues, the expression of *AKR1C2* and *AKR1C1* genes in prostate cancer samples has 9 selective reductions, while the expression of *AKR1C3* genes is not selectively reduced [36]. Pipione et al. [37] reported that *AKR1C3* gene plays an important role in AR synthesis and is a potential target for the treatment of castrated prostate cancer. Hara et al. [38] reported that *AKR1C1*, *AKR1C2*, *AKR1C3* can mediate the metabolism of fatty acids in the conductor, and negatively regulated by the level of free unsaturated fatty acids, its overexpression is related to the pathogenesis of extrahepatic cancer. Based on the above research and the results of this study, it is speculated that AKR1 family gene may affect TAM resistance by participating in the metabolism of estrogen and androgen, but the specific mechanism needs to be further verified.

Le et al. [39] reported that *AKR1C1* and *AKR1C2* proteins were involved in the process of cancer cell resistance, and selective targeting of GLUT-3 in the *AKR1C* protein of brain glioma can delay the acquisition of drug resistance temozolomide in astrocytes. In order to verify the expression and function of hub gene in TAM resistance, we download another data - microarray data GSE67916 to verify the genes of *STAT1*, *ESR1*, *FN1*, *CYP1B1*, *AKR1C1*, *AKR1C2*, *AKR1C3* and *UGT1A* family, the results showed that only *AKR1C1*, *AKR1C3* and *UGT1A6* were significantly higher in TamR group than those in MCF7/S0.5 group. It confirmed that AKR1 family gene may participate in the process of TAM resistance. The prognosis of cancer resistant patients was generally poorer, therefore we downloaded breast cancer dataset in the TCGA database and analyzed the relationship between the previously screened hub gene and prognosis. The previous results showed that AKR1 family gene was most likely to participate in the process of TAM resistance. Therefore we knockdown or overexpression the *AKR1C1, AKR1C2 or AKR1C3,* the results showed that in sensitive breast cancer cell lines, overexpression of the *AKR1C1*, *AKR1C2* or *AKR1C3* can significantly increase the cell TAM EC50 value; and in breast cancer resistant cell lines, knocking out *AKR1C1*, *AKR1C2* or *AKR1C3* can significantly reduce the cell TAM EC50 value. Fifty nanometer TMA was used to treat breast cancer sensitive cell lines overexpressing *AKR1C1*, *AKR1C2* or *AKR1C3*, its proliferation activity was significantly higher than the wild type. Five hundred nanometer TAM was used to treat breast cancer TMA-resistant cells that knocked out *AKR1C1*, *AKR1C2* and *AKR1C3* lines, its proliferation activity was significantly lower than the wild type, which directly indicated that high *AKR1C1*, *AKR1C*2 or *AKR1C3* gene expression promoted TAM resistance.

## Conclusion

In this study, we used a variety of bioinformatics analysis tools to explore the differentially expressed genes that affect of TAM resistance in the early stage with invasive lobular breast cancer, and preliminarily screened out AKR1 family gene and the mechanism of resistance, so as to provide a theoretical reference for the clinical treatment of invasive lobular breast cancer. However, there are still some deficiencies in this study. Firstly, the data in this study are from public databases, which can not ensure the data quality; secondly, the sample size is small, which may have some bias; finally, our results are still lack of clinical experimental verification, so we need to carry out corresponding clinical experiments to verify our results later.

## Supplementary Information

**Additional file 1.**

**Additional file 2.**

## Data Availability

All data are available via the corresponding author. The datasets analysed during the current study are available in the Gene Expression Omnibus (GEO) database, numbered GSE96670. (https://www.ncbi.nlm.nih.gov/geo/query/acc.cgi?acc=GSE96670).

## Abbreviations

AKR1: Aldo-keto reductase family 1
TAM: Tamoxifen
ER: Estrogen receptor
TCGA: The Cancer Genome Atlas
GEO: Gene expression omnibus
PPI: Protein-protein interaction
STAT1: Signal transducer and activator of transcription 1
ESR1: Estrogen receptor alpha
FN1: Fibronectin1
CYP1B1: Cytochrome P4501B1
UGT: Uridine diphosphate glucuronosyltransferase
