# Supplementary material for: The role of AKR1 family in tamoxifen resistant invasive lobular breast cancer based on data mining
Source: BMC Cancer. 2021 Dec 9;21:1321. doi: 10.1186/s12885-021-09040-8 (PMC8662825; doi:10.1186/s12885-021-09040-8)
Supplement: Supplementary file 2 — Additional file 2. [file 12885_2021_9040_MOESM2_ESM.doc]

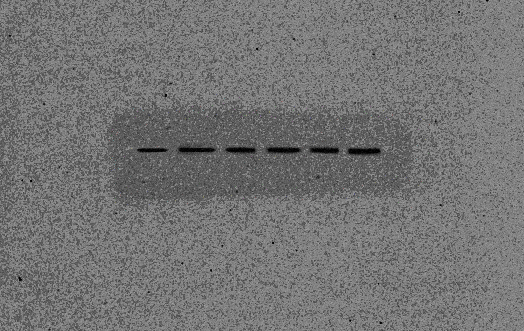


AKR1C1 with exposed for 2 h


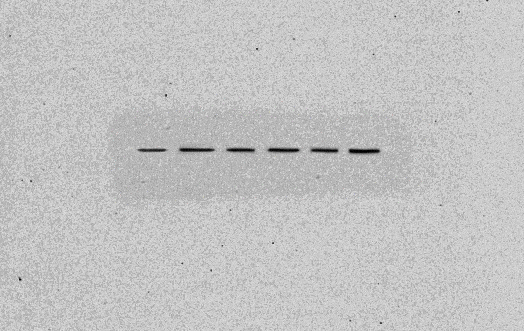


AKR1C1 with exposed for 6 h


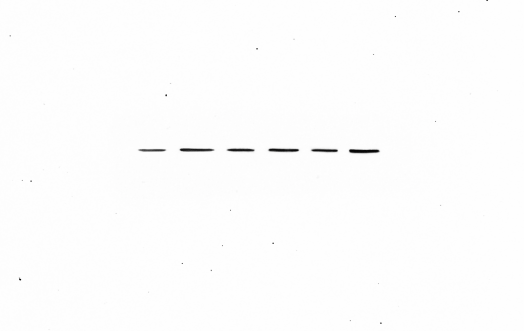


AKR1C1 with exposed for 12 h


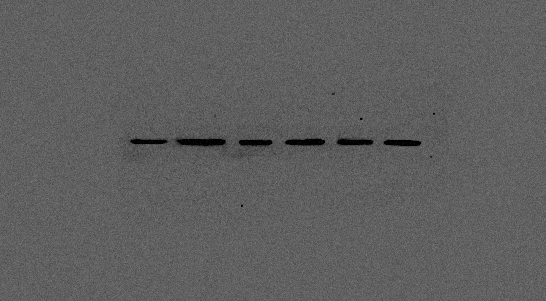


AKR1C2 with exposed for 2 h


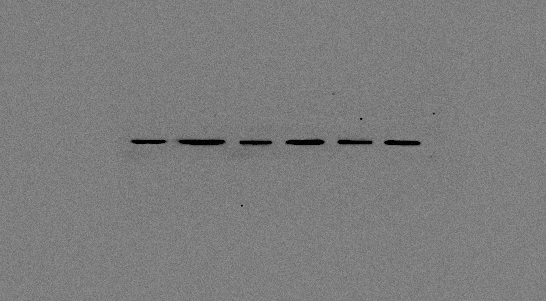


AKR1C2 with exposed for 4 h


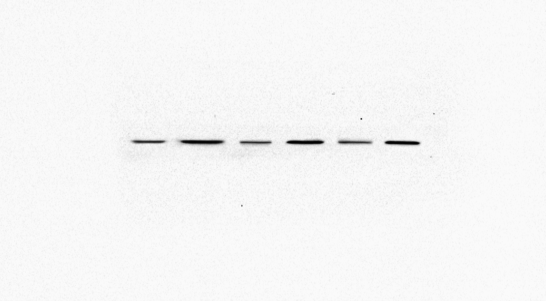


AKR1C2 with exposed for 12 h


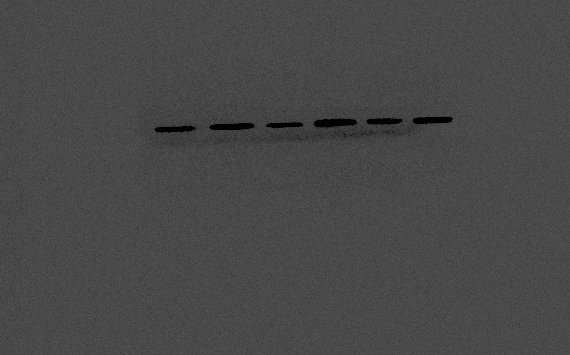


AKR1C3 with exposed for 1 h


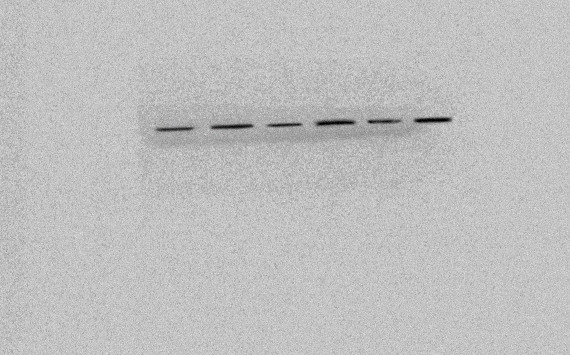


AKR1C3 with exposed for 8 h


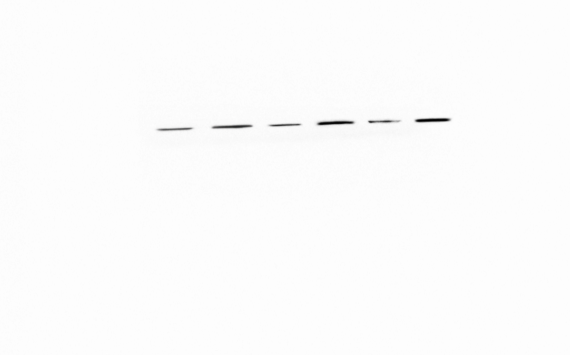


AKR1C3 with exposed for 12 h


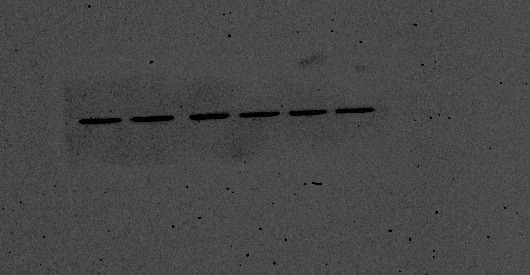


GAPDH with exposed for 1 h


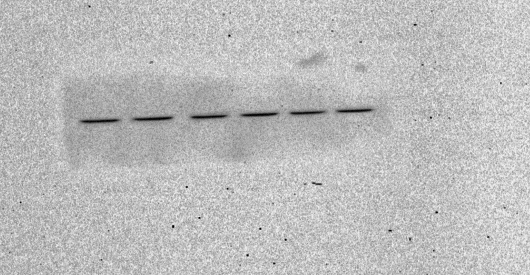


GAPDH with exposed for 6 h


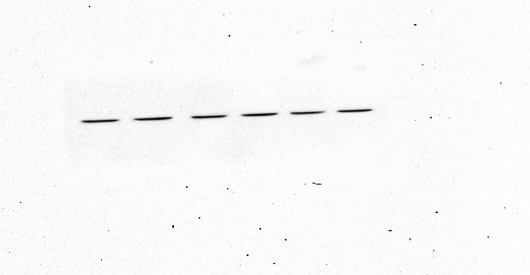


GAPDH with exposed for 12 h
